# Quantitative Non-Linear Effect of High Ambient Temperature on Chloride Threshold Value for Steel Reinforcement Corrosion in Concrete under Extreme Boundary Conditions

**DOI:** 10.3390/ma14247595

**Published:** 2021-12-10

**Authors:** Abdulrahman M. Alhozaimy, Mshtaq Ahmed, Raja Rizwan Hussain, Abdulaziz Al-Negheimish

**Affiliations:** 1 Center of Excellence for Concrete Research and Testing (CoE-CRT), Civil Engineering Department, College of Engineering, King Saud University, Riyadh 11421, Saudi Arabia; alhozimy@ksu.edu.sa (A.M.A.); negaimsh@ksu.edu.sa (A.A.-N.); 2 Civil Engineering Department, College of Engineering, King Saud University, Riyadh 11421, Saudi Arabia; mshahmed@ksu.edu.sa

**Keywords:** chloride threshold value, temperature, corrosion, steel reinforcement bar, concrete

## Abstract

This paper investigates the effect of high ambient temperatures on the chloride threshold value for reinforced concrete (RC) structures. Two commonly available carbon steel rebars were investigated under four different exposure temperatures (20 °C (68 °F), 35 °C (95 °F), 50 °C (122 °F), and 65 °C (149 °C)) using environmental chambers at a constant relative humidity of 80%. For each temperature, six different levels of added chloride ions (0.00%, 0.15%, 0.30%, 0.60%, 0.90%, and 1.20% by weight of cement) were used to study the chloride threshold value. Corrosion initiation was detected by monitoring the corrosion potential and corrosion rate using electrochemical techniques. The water-soluble (free) and acid-soluble (total) chlorides were determined using potentiometric titration according to the relevant ASTM standards. The threshold chloride content for each exposure temperature was determined by analyzing the corrosion potential, corrosion rate, and chloride content of each specimen. The results showed that the chloride threshold values were significantly temperature-dependent. At temperatures of 20 °C (68 °F) and 35 °C (95 °F), the chloride threshold value (expressed as free chlorides) was approximately 0.95% by weight of cement. However, as the temperature increased to 50 °C (122 °F), the chloride threshold decreased significantly to approximately 0.70% by weight of cement. The reduction in the chloride threshold value became more dramatic at an exposure temperature of 65 °C (149 °F), decreasing to approximately 0.25% by weight of cement. The trends were similar for the rebars from the two sources, indicating that the rebar source had little influence on the chloride threshold value.

## 1. Introduction

The chloride-induced corrosion of reinforced concrete (RC) structures is one of the most important deterioration mechanisms affecting their durability and useful service life. The mechanisms and impact of reinforcement corrosion on the durability of RC are well known. The steel reinforcement in concrete is protected, initially, by a thin layer formed on the surface of the rebars called the passive layer [1]. This protective film is maintained by the alkaline environment of the concrete. However, if this passive layer is destroyed by the attack of chloride ions, the chloride-induced corrosion of the reinforcement will begin. This corrosion will continue as long as supplies of both moisture and oxygen are available. The amount of chloride needed to break the passive layer and hence initiate the corrosion is called the chloride threshold (CT) value. Schiessl and Raupach [2] defined the CT value as the content of chloride at the steel depth that is necessary to sustain local passive film breakdown and hence initiate the corrosion process.

The CT value is an important parameter in studying the phenomenon of steel corrosion in RC structures. However, despite the numerous studies reported in the literature, there is no specific number that can be assigned to this value, and more precise values are still needed [3,4]. The issue is complicated by the fact that the CT value is represented in the literature using more than one expression. It can be presented as the ratio of Cl^−^/OH^−^ in a concrete pore solution or as the total chloride (acid-soluble chloride) or free chloride (water-soluble chloride) by weight of cement [5]. The first measurement of the CT value was reported by Hausmann [6] using a synthetic concrete solution with a [Cl^−^]:[OH^−^] ratio of 0.6. A wide range of CT values are presented in the literature, including Cl^−^/OH^−^ ratios ranging from 0.12 to 3.0, 0.04–2.42% total chloride by weight of cement, and 0.03–4% free chloride by weight of cement [7]. The considerable spread of CT values encountered in the literature may be the result of the high number of variables that influence it, with many of these factors being interrelated [3,7,8].

Limiting the critical chloride content in concrete codes and standards has been a key durability design requirement for RC structures for many years. In the USA, the ACI Building Code Requirements [9] limit the water-soluble chloride (free chloride) content by mass of cement to 0.15% for RC exposed to moisture and an external source of chloride and 0.3% for RC exposed to moisture but not to an external source of chloride, with a limit of 0.06% for prestressed concrete structures regardless of the exposure conditions. The British Standard [10] limits the total chloride content to less than 0.4% for RC structures and 0.1% for prestressed concrete structures. The value of 0.4% for the total chloride ions by weight of cement is also considered by RILEM to be an appropriate threshold [11].

Recently, the importance of accurately assessing the CT value has been heightened because it is a key parameter in predicting the service life of structures in chloride environments [12,13,14]. One possible definition of the service life of a structure is the time required for transport processes to increase the chloride level at the depth of the steel to the CT value. Conservative values such as 0.2% or 0.4% by weight of cement have been used to predict the corrosion-free life because of the uncertainty regarding the actual limits for chloride-induced corrosion in various environments [15,16,17,18,19].

Temperature has long been recognized in the literature as a major factor with a significant effect on corrosion [3,20,21]. However, recently published works on the influence of temperature on corrosion provide a more complex picture. Sharifi-Asl et al. [22] found that the corrosion resistance of carbon steel in a 12.5 pH saturated Ca(OH)_2_ solution decreased with increasing temperature from 25 °C (77 °F) to 85 °C (185 °F). On the other hand, Lu et al. [23] found that carbon steel did not exhibit pitting corrosion at room temperature in simulated concrete pore water (pH = 13.5) without chloride ions, regardless of the temperature. It is also evident from reviewing the literature that studies on the effect of temperature on the CT value of carbon steel are limited, with contradictory outcomes. Hussain et al. [24] investigated the effect of the exposure temperature on the CT value and concluded that the exposure temperature has a very strong influence on CT values. They reported that an increase in temperature from 20 °C (68 °F) to 70 °C (158 °F) caused a 5-fold reduction in the CT value. On the other hand, Matsumuraa et al. [25] studied the effect of temperature on corrosion in RC specimens under high temperatures in the range of 65–90 °C (149–194 °F) and found that increasing the exposure temperature from 65 °C (149 °F) to 90 °C (194 °F) led to an increase in the CT value. Furthermore, a recent investigation of the effect of temperature on the corrosion of stainless steel rebars in concrete [26] showed a remarkable reduction in the CT value with increasing temperature for low-nickel duplex stainless steel but not for traditional austenitic stainless steels. Therefore, there is an obvious need to carry out a comprehensive investigation of the effects of the temperature, chloride content, and rebar sources on the corrosion of RC structures to clarify and supplement existing knowledge. This paper reports on the results of a comprehensive investigation of the effect of high exposure temperatures on the CT value for RC structures. This paper proposes CT values for various exposure temperatures, which can be the basis of future modifications to code limits to cover hot climates.

## 2. Research Significance

The amount of chloride required to initiate corrosion (CT value) is important in the design of RC structures against corrosion attacks. The CT value is also an essential input for service life models. However, the influence of exposure ambient temperature is not yet considered when specifying the CT value. This research showed that the CT value is temperature-dependent and must be considered for effective and more realistic assessment/design against reinforcement corrosion in tropical climates. The maximum temperature in this investigation was kept at 65 °C as the ambient temperature in some hot countries can exceed 50 °C. In those regions, the surface temperature of concrete can reach 65 °C under direct exposure to the sunlight during the summer season. This paper proposes CT values for various exposure temperatures, which can be the basis of future assessment/modifications to code limits for covering hot climates.

## 3. Experimental Program

### 3.1. Materials and Mix Proportions

Type I ordinary Portland cement, in compliance with the requirements of ASTM C150, was used. Crushed limestone with a maximum size of 10 mm (0.4 in.) was obtained from quarries around Riyadh, Saudi Arabia, and used as coarse aggregate. The fine aggregate was a blend of natural white sand and crushed limestone that satisfied the ASTM C33 limits. Natural sand available in the gulf region is too fine. Therefore, crushed sand is used to make the fine aggregate well graded. This is a common practice in Saudi Arabia and the gulf region. The proportions of the concrete mix used in this study are listed in Table 1. The mix had a w/c ratio of 0.50, which is the maximum limit permitted by ACI 318 [9] for durability.

Deformed steel rebars with a diameter of 14 mm (0.55 in.) provided from two different popular suppliers in Saudi Arabia were used in this study. The chemical compositions of these steel bars are listed in Table 2. The steel rebars from Source A were manufactured using an electric arc furnace (EAF) based on the thermo-mechanical treatment method (TMT). The microstructure of these rebars consisted of pearlite-ferrite at the core and tempered martensite at the rim. The rebars from Source B were alloy steel produced by the normal heating of rolls without quenching and tempering treatment.

### 3.2. Specimen Preparation and Curing Conditions

**Figure 1:**
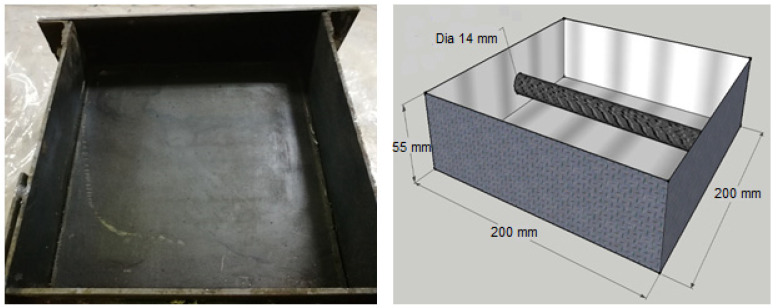
Steel mold used to cast prismatic specimens.

Prismatic-reinforced concrete specimens with the dimensions of 200 mm × 200 mm × 55 mm (7.87 in. × 7.87 in. × 2.17 in.) were cast to monitor the corrosion potential and corrosion rate under the four investigated temperatures. Companion plain concrete specimens were cast from the same concrete mixture to measure the water-soluble and acid-soluble chlorides for every level of added chloride ions. Figure 1 shows a schematic diagram and an original image of the prismatic steel mold used to cast the specimens.

**Figure 2:**
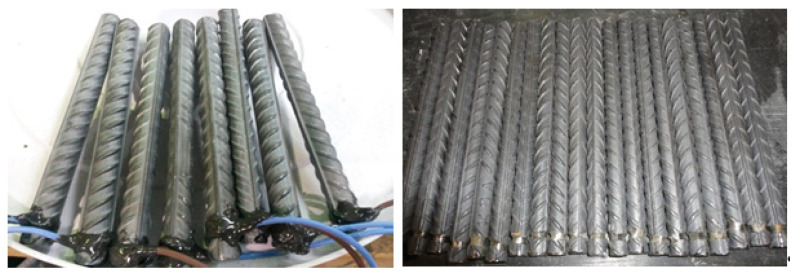
Rebar samples (14 mm diameter) connected by electric wires.

The rebar samples were cut to a length of 16 cm (6.3 in.) and fully embedded in the concrete. Electrical contact with the steel bar was achieved using a copper wire that was effectively glued to the rebar using epoxy. Grooves were made around the end of the rebar to ensure good contact between the copper wire and rebar. Figure 2 shows the rebar samples and electric contact made by an electric copper wire. A clear cover of 20 mm (0.8 in.) was used outside the bar because the measured half-cell potential values at the specimen surface could be considered to be the actual value at the steel surface if the cover depth was within 20 mm (0.8 in.) [27]. Therefore, the maximum aggregate size was limited to 10 mm (0.4 in.) in this study. It is well documented in the past literature that the coarse aggregate size is not a significant parameter for corrosion investigation under consideration.

**Figure 3:**
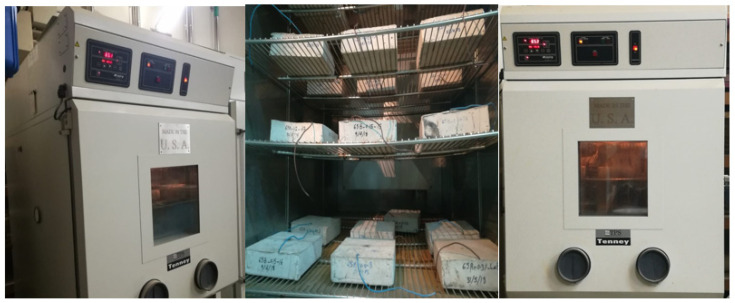
Environmental chambers were used to maintain specimens at desired temperature and humidity for the duration of the testing period.

The introduction of chloride into the concrete was done by adding chloride into the mixing water of the concrete. Sodium chloride (NaCl) with 99.5% purity was used as the source for the chloride ions, which were added to fresh concrete at six different levels (0.00, 0.15, 0.30, 0.60, 0.90, and 1.20%) by mass of cement. Two replicate concrete specimens were cast for each chloride level. After de-molding, the specimens were moist cured under laboratory conditions for 28 days and then exposed to four different temperatures (20 °C (68 °F), 35 °C (95 °F), 50 °C (122 °F), and 65 °C (149 °F)) with a constant relative humidity of 80% for the entire testing period using four environmental chambers (Figure 3). The pH value of the concrete was 12.4 which is within the normal range. It was measured using Jenway pH Meter. Since the chloride threshold value also varies with the pH of concrete, it was necessary to mention the pH of concrete even if it was not a variable in the experimentation.

### 3.3. Corrosion Measurement

The corrosion potential and corrosion rate of the steel rebars were measured using a commercial corrosion meter. The GECOR measures the corrosion rate as reflected by the corrosion current density and half-cell corrosion potential. Corrosion rate measurement usually involves the application of an electrical signal through a connection to the steel bar. In the GECOR method, this signal is confined to the steel rebar in a circle with a diameter of 110 mm (4.33 in.). There is evidence that this technique provides a more accurate measurement of the corrosion rate [28]. It was well established by Stern and Geary [29] that the corrosion current is linearly related to the polarization resistance, which provides a direct quantitative measurement of the amount of steel turning into oxide at the time of measurement. The corrosion current values in the GECOR [30] are calculated from polarization resistance R_p_ using the relation I_corr_ = B/R_p_, where I_corr_ is given in µA/cm^2^ when R_p_ is given in kΩ/cm^2^ and B = 26 mV.

### 3.4. Chloride Content

Two types of chlorides were analyzed: water-soluble and acid-soluble. Samples were prepared for the water-soluble and acid-soluble chlorides according to ASTM C1218 [31] and ASTM C1152 [32], respectively. Concrete samples were taken from the plain concrete specimens by drilling the specimens at different locations to get fine powder. The powder samples were then dissolved, heated, and filtered through filter paper using a filtration machine. Finally, the chloride ions in the prepared solutions were determined by potentiometric titration with the help of titration equipment using silver nitrate as a titrant.

The reinforced specimens are not broken so far as they are still subjected to another phase of measurement. The extraction of rebars for physical examination, mass loss measurements, microstructural investigations, chemical analysis of rust collections, SEM, XRD, XRF, Raman etc. shall all be carried out at a later stage and published afterwards.

## 4. Results and Discussion

### 4.1. Chloride Content Analysis

The acid-soluble and water-soluble chlorides are summarized in Table 3 for every level of chloride ions added to the concrete mixture. The results for the water-soluble and acid-soluble chlorides listed in Table 3 are the average of two powder samples taken from two plain concrete specimens at the age of 28–40 days. The values obtained from every two samples are very similar. The maximum difference between two samples does not exceed 7% of the largest value, which is in a well acceptable range for corrosion experiments. The presence of chlorides in the zero-added-chloride mixture was due to the presence of residual chloride ions in the various ingredients of concrete. The difference between the acid-soluble and water-soluble chloride values was attributed to chloride that was chemically combined in hydration reaction products, which is called bound chloride. As listed in Table 3, the bound chloride was 30% at the zero level of added chloride, and it decreased to approximately 13% as the amount of chloride added to the concrete mix increased. When expressed as a percentage of the acid-soluble (total) value, the water-soluble chloride (free) ranged from 70% to 88%. This ratio was in the higher range found in the literature. Research at the Federal Highway Administration found that the conversion factor from acid-soluble to water-soluble chloride could range from 0.35 to 0.90, depending on the constituents and history of the concrete [18]. In other studies, water-soluble chloride in the range of 50% to 85% of the acid-soluble chloride was reported, with the remainder chemically combined in hydration reaction products [33,34,35]. 

### 4.2. Corrosion Potential

The half-cell potential is a universally accepted standard test used to assess the state of embedded steel reinforcement, specifically for any corrosion activity. According to ASTM C876 [36], if potentials over an area are more positive than −200 mV CSE, there is a greater than 90% probability that no reinforcing steel corrosion is occurring in that area at the time of measurement. If the potentials over an area are in the range of −200 to −350 mV CSE, the corrosion activity of the reinforcing steel in that area is uncertain. If the potentials over an area are more negative than −350 mV CSE, there is a greater than 90% probability that reinforcing steel corrosion is occurring in that area at the time of measurement.

**Figure 4:**
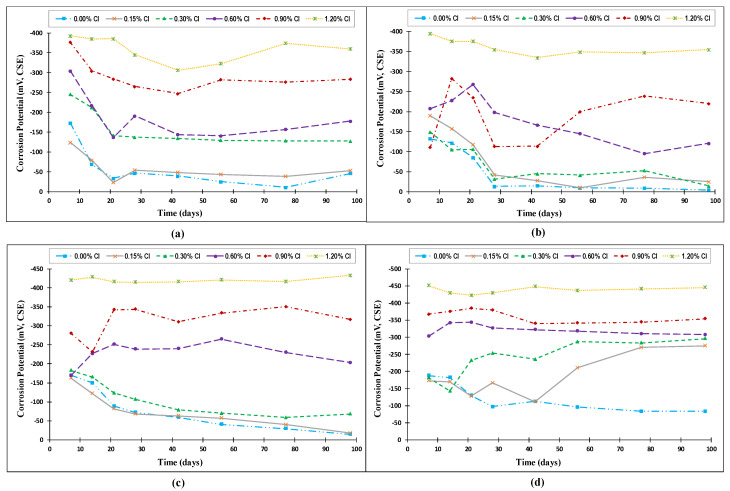
Variation of corrosion potential with time for specimens exposed to temperatures of: (**a**) 20 °C (68 °F), (**b**) 35 °C (95 °F), (**c**) 50 °C (122 °F), and (**d**) 65 °C (149 °F) (Source A).

Figure 4 shows the variation in the corrosion potential with time for steel rebars exposed to the four investigated temperatures (20 °C (68 °F), 35 °C (95 °F), 50 °C (122 °F), and 65 °C (149 °F)). Generally, the behaviors of the corrosion potential over time for all specimens exposed to the four temperatures could be divided into two general trends. In the first trend, the corrosion potential started with high values at the early age of the specimens and decreased with time until it stabilized, with only limited fluctuations. The corrosion potential in the second trend varied significantly with time at the early age of the specimens, and the variation decreased with time.

**Figure 5:**
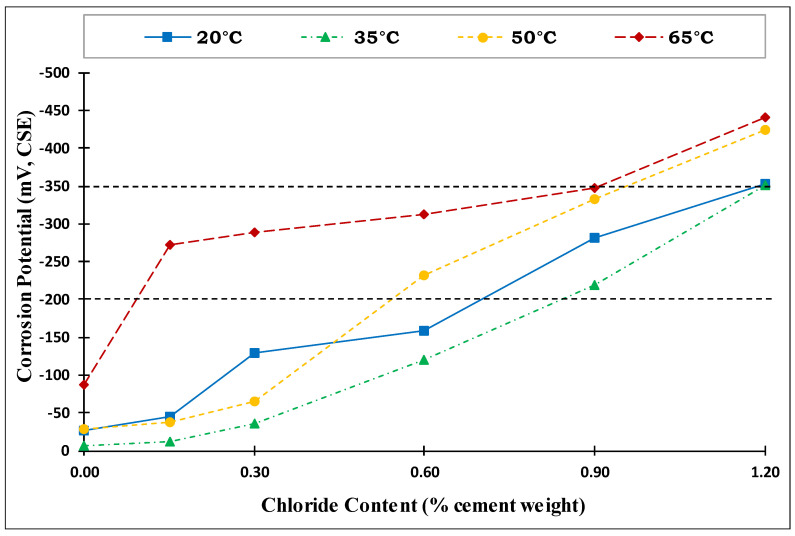
Corrosion potential vs. added-chloride content for different exposure temperatures (Source A).

The corrosion potential based on the average of the last three measurements for the four exposure temperatures is plotted versus the chloride content in Figure 5. As shown in this figure, the corrosion potential increased non-uniformly with the chloride content. The corrosion potential for specimens exposed to a temperature of 35 °C (95 °F) was slightly less than the corrosion potential of specimens exposed to 20 °C (68 °F). This indicated that the temperature had no significant effect on the corrosion potential at low temperatures until 35 °C (95 °F). As the temperature increased to 50 °C (122 °F), the corrosion potential increased slightly for specimens with low-added chlorides (0.0% to 0.3%). However, the increase in corrosion potential was more significant for specimens with high-added chlorides (0.6% to 1.2%). At a temperature of 65 °C (149 °F), all the specimens showed high corrosion potential values, except those with zero-added chlorides.

### 4.3. Corrosion Rate

The corrosion rate of the steel reinforcement in concrete is an important parameter that can be used to assess the corrosion activity. According to the RILEM criteria listed in Table 4 [37], when the corrosion intensity is less than 0.1 µA/cm^2^, the steel is considered to be in a passive condition. Gonzalez and Andrade [38] reported that the corrosion rate of reinforcing steel is often regarded as significant when it exceeds 0.1–0.2. µA/cm^2^. Alonso et al. [39] noticed that specimens with corrosion rate values greater than 0.1 µA/cm^2^ showed visible corrosion. Therefore, a value of 0.1 μA/cm^2^ was adopted in this research to consider the initiation of corrosion in steel rebars embedded in concrete. The same specimens have been used over the course of time for corrosion potential and corrosion current measurements. It makes the measurements more reliable and reproducible adding authenticity and reliability to the paper. The measurements are instantaneous using D.C technique, which does not influence the specimen much over the course of time. Furthermore, there was no impressed current or accelerated corrosion measurements that may influence the same specimens with time.

**Figure 6:**
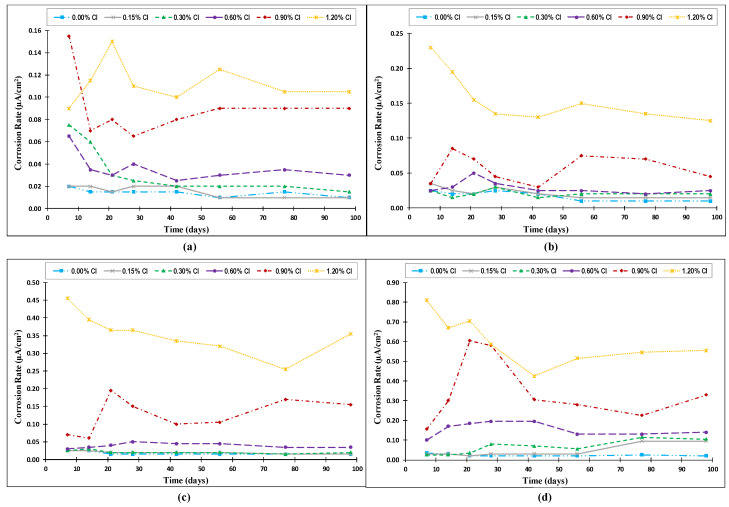
Variation of corrosion rate with time for specimens exposed to temperatures of: (**a**) 20 °C (68 °F), (**b**) 35 °C (95 °F), (**c**) 50 °C (122 °F), and (**d**) 65 °C (149 °F) (Source A).

The variations of the corrosion rate with time for steel rebars exposed to the four investigated temperatures (20 °C (68° F), 35 °C (95 °F), 50 °C (122 °F), and 65 °C (149 °F)) are presented in Figure 6 for a 100-day duration. For specimens exposed to a temperature of 20 °C (68 °F), the corrosion rate had relatively high initial values at an early age for all the specimens, and then decreased with time until it eventually stabilized. At an exposure temperature of 35 °C (95 °F), the variation in the corrosion rate was small at an early age for specimens with 0.0–0.6% added chlorides, and it decreased with time until it stabilized. Specimens with 0.9–1.2% added chlorides had greatly varying corrosion rates at an early age, but they tended to stabilize at a later age for the specimens. The specimens exposed to a temperature of 50 °C (122 °F) had two different trends for their corrosion rate behaviors. The first one was for specimens with low-added chlorides (0.0–0.6%), which showed almost no variation with time for the entire period of corrosion monitoring. The second one was for specimens with high-added chlorides (0.9–1.2%), which varied significantly with time at an early age, and the variation decreased with time. For the specimens exposed to 65 °C (149 °F), three trends for their corrosion rate behaviors were observed. The first one was for specimens with zero-added chlorides, which had a constant corrosion rate. The second one was for specimens with higher values of added chloride (0.6–1.2%). The variation in the corrosion rate for these specimens was high at the early age of the specimens, and it decreased over time as the CSH gel stabilized, permeability and moisture content reduced, etc. The third trend was for specimens with 0.15–0.3% added chlorides, which remained constant with time for the first two months, then increased until it stabilized again. This trend was not observed for the other lower temperatures. It can be said that activation energy produced at high temperature of 65 °C (149 °F) contributed to the breakdown of the passive layer after a longer period of exposure compared to that of a higher level of chlorides.

Overall, there was an instability of the electrochemical process for the rebars as well as the passive film at the early age of concrete specimens due to the premature formation of the protective layer. The variation in the CSH gel, moisture concentration, capillary pores connectivity, tortuosity etc. all are premature at the initial stage and can cause sudden changes in the potential, as this is an instantaneous value taken at that particular interval of time. As the time progresses, the CSH gel stabilizes, permeability reduces, moisture content reduces, and pores get disconnected etc., causing an overall reduction in the corrosion rate.

**Figure 7:**
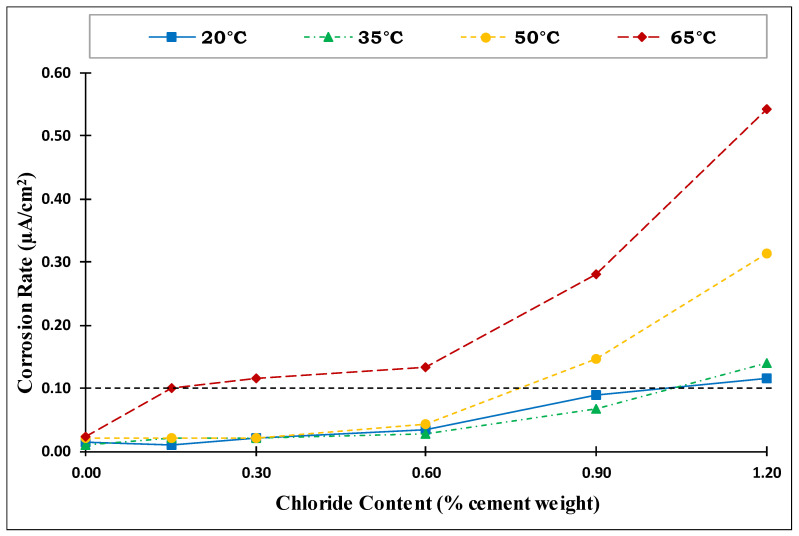
Corrosion rate vs. added chloride content at different temperatures (Source A).

Figure 7 shows the corrosion rate vs. the chloride content at the four investigated temperatures. The average of the last three readings recorded after the stabilization of the corrosion rate was used to establish this chart. Generally, the corrosion rate behavior shown in Figure 7 was similar to the behavior of the corrosion potential. The figure shows that the corrosion rate values for the specimens exposed to temperatures of 20 °C (68 °F) and 35 °C (95 °F) were comparable for all the different chloride contents. This confirmed the results obtained from the corrosion potential, showing that the temperature had no significant effect on the corrosion at low temperatures until 35 °C (95 °F). As the temperature increased to 50 °C (122 °F), the corrosion rate for specimens with low-added chlorides (0.0–0.6%) was similar to those for specimens exposed to 20 °C (68 °F) and 35 °C (95 °F). However, the corrosion rate for specimens with high-added chlorides (0.9–1.2%) increased significantly compared to those at temperatures of 20 °C (68 °F) and 35 °C (95 °F). At an exposure temperature of 65 °C (149 °F), the specimens with the various added-chloride contents (0.15, 0.3, 0.6, 0.9, and 1.2%) all showed significant increases in the corrosion rate.

**Figure 8:**
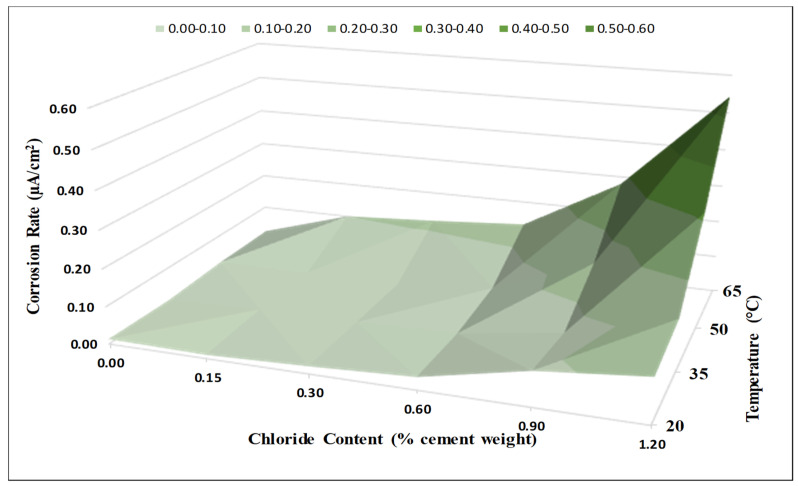
Coupled effects of chloride content and temperature on corrosion rate (Source A).

The combined influence of the chloride content and temperature on the corrosion rate is illustrated in Figure 8. As shown in this figure, the corrosion rates with 0.9% and 1.2% added chloride contents increased for all the investigated temperatures. However, the increase in the corrosion rate was more significant at high temperatures. At a temperature of 65 °C (149 °F), the increase in the corrosion rate became significant for all the added chloride contents (0.15%, 0.3%, 0.6%, 0.9%, and 1.2%). The figure clearly indicates strong coupled effects of chloride content and exposure temperature on corrosion rate. The specimens are now subjected to another phase of measurement. The extraction of rebars for physical examination, mass loss measurements, microstructural investigations, chemical analysis of rust collections, SEM, XRD, XRF, Raman etc. shall all be carried out at a later stage and published afterwards.

### 4.4. Effect of Rebar Sources

**Figure 9:**
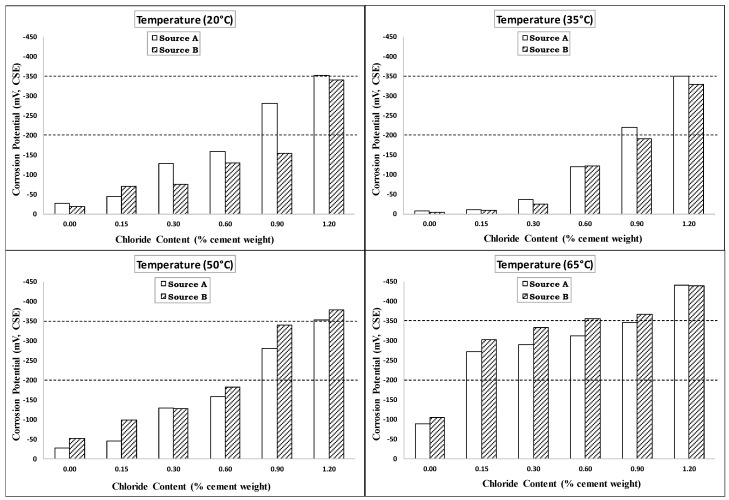
Comparison of corrosion potentials for Source A and Source B rebars at different exposure temperatures.

**Figure 10:**
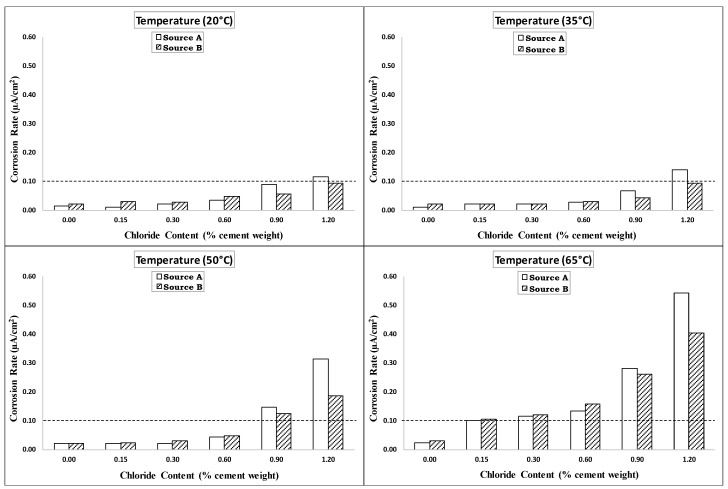
Comparison of corrosion rates for Source A and Source B rebars at different exposure temperatures.

Comparisons of the corrosion potentials of the two sources of steel rebars at the four investigated temperatures are presented in Figure 9. As shown in this figure, the corrosion potentials for the two rebar sources had comparable values at all the added chloride contents for all the exposure temperatures except for the 0.9% added chloride content at a temperature of 20 °C (68 °F). Figure 10 shows comparisons of the corrosion rates for the two sources of steel rebars at the four investigated temperatures. As shown in this figure, the steel rebars from Source A had similar corrosion rates to those from Source B except for those with the high added-chloride content of 1.2% and at high exposure temperatures of 50 °C (122 °F) and 65 °C (149 °F). This means that in a severe environment with high temperature and high chloride content, the rebar source had an effect on the corrosion rate. However, the effect of the rebar source on the CT value was marginal. Generally, it can be said that the trends for the corrosion potential and corrosion rate for all the specimens reinforced with steel rebars from Source A were similar to those of the specimens reinforced with rebars from Source B at the four investigated temperatures.

### 4.5. CT Values

**Figure 11:**
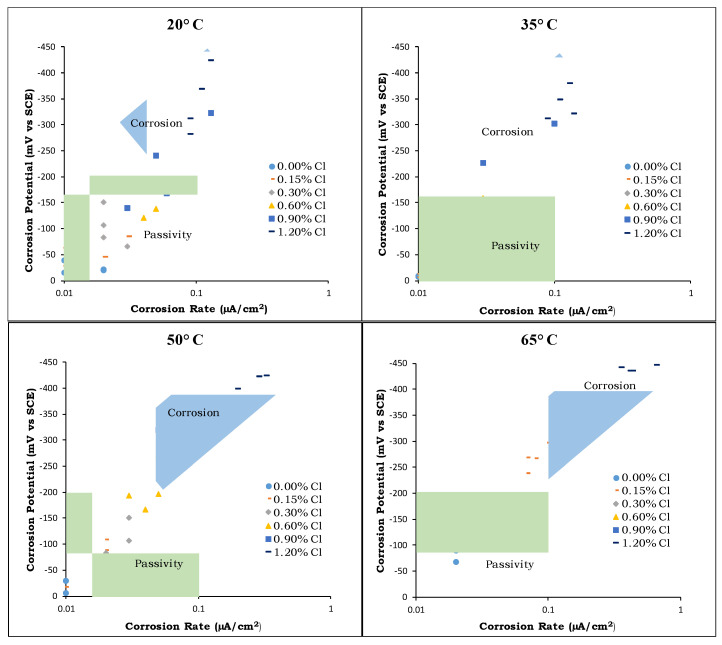
Tafel’s diagram (potential vs. current) for rebar corrosion initiation.

Figure 11 shows the Tafel’s diagram relationship between the potential and current for corrosion initiation. In this figure, all the data for both rebar sources (A and B) are plotted for the four investigated temperatures. The area of passive steel is characterized by a corrosion potential of less than −200 mV vs. CSE and a corrosion rate lower than 0.1 µA/cm^2^. It is clear from the figure that a significant interaction between the temperature and chloride content exists with respect to depassivation. For the specimens with insignificant chloride content (0% added chlorides), the passivity of the rebars was maintained even at 65 °C (149 °F). This was consistent with the findings of Lu et al. [23], who reported that carbon steel exhibited no corrosion when tested in simulated concrete pore water (pH = 13.5) without chloride ions, regardless of the temperature. As the chloride content increased, the behavior became more temperature-dependent. At temperatures of 20 °C (68 °F) and 35 °C (95 °F), most of the test measurements carried out on specimens from both rebar sources showed results in the passivity zone. However, as the temperature increased, the measured values moved into another zone (labeled as corrosion in Figure 11). At 50 °C (122 °F), the measurements for rebars in concrete with 0.6%, 0.9%, and 1.2% added-chloride contents lay in the corrosion zone. At 65 °C (149 °F), all the measurements, except for that of the 0% added chloride, fell in this zone, illustrating the crucial role of temperature on the de-passivation of steel rebars. It should be noted that the standard AENOR UNE 83992–2 [40] stipulates that Ecorr values less than or equal to −300 mV and Icorr values greater than or equal to 0.1 µA/cm^2^ are indicative of de-passivation.

**Figure 12:**
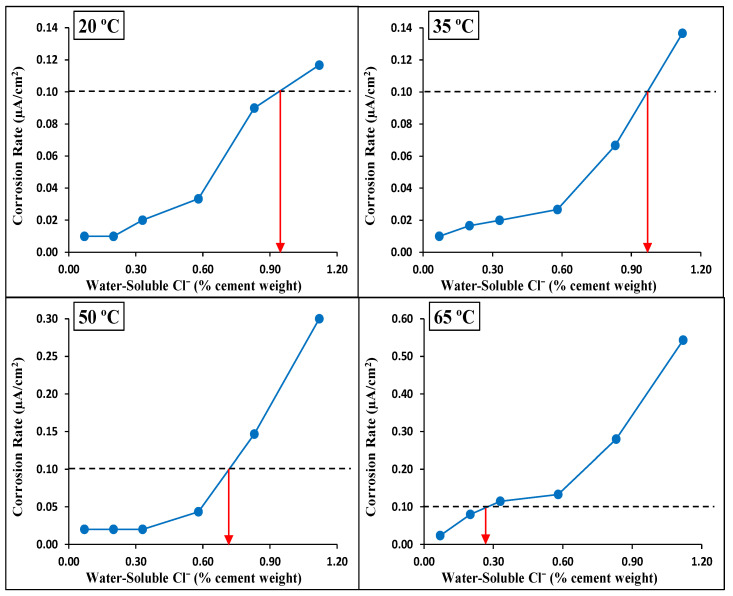
Determination of CT value (water-soluble chlorides by weight of cement) based on corrosion rate for different exposure temperatures (Source A).

Figure 12 identifies the CT values for the four investigated temperatures based on the corrosion rate. In this figure, the average corrosion rate for each set of specimens is plotted versus the water-soluble chloride ions in the concrete. The CT values were identified for the four investigated temperature conditions corresponding to a corrosion rate of 0.1 µA/cm^2^, which was used as a criterion to distinguish between the passivation and de-passivation of the steel rebars. A similar exercise was carried out to identify the CT values from corrosion potential measurements based on the ASTM C876 [36] criteria. The water-soluble CT values for both sources (A and B), as determined based on the corrosion potential and corrosion rate measurements, are summarized in Table 5. It can be seen that the CT values based on the corrosion potential have a wide range, while those based on the corrosion rate have specific values. This is expected because of the uncertainty associated with the initiation of de-passivation based on corrosion potentials compared to the deterministic approach based on the corrosion rate. However, it can be seen that the CT values obtained from the corrosion rate are consistently within the range of the corresponding CT values obtained from the half-cell potential. This provides good confidence in the reliability of the CT values obtained from corrosion rate measurements based on the 0.1 µA/cm^2^ criterion.

**Figure 13:**
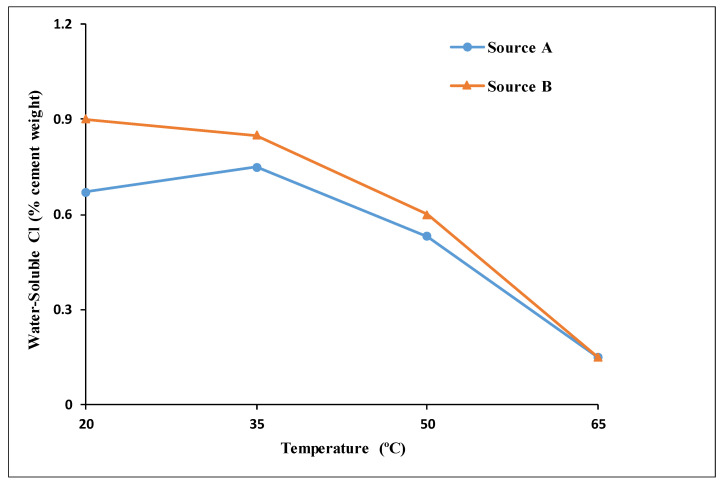
CT value vs. temperature for rebar from both sources (A & B) based on corrosion rate.

The water-soluble CT values for specimens from both sources (A and B), as determined based on the corrosion rate measurements, are compared in Figure 13. The different sources showed similar decreasing trends for the CT values with an increase in the exposure temperature, highlighting the higher risk of corrosion for steel reinforcements in concrete under hot weather. Above 35 °C (95 °F), the CT values for the specimens from both sources decreased almost linearly with an increase in the exposure temperature. At an exposure temperature of 65 °C (149 °F), the CT values were almost the same (0.25% vs. 0.26% by weight of cement), as both sources registered a dramatic drop to approximately 1/4 of the CT value at 20 °C (68 °F), clearly highlighting the crucial influence of high temperature on the de-passivation of carbon steel rebars, regardless of the source. The magnitude of the drop in the CT value due to an increase in temperature from 20 °C (68 °F) to 65 °C (149 °F) was similar to that reported by Hussain et al. [24]. This effect and temperature dependency were highly significant because the CT values at 65 °C (149 °F) were lower than the chloride limit specified in the internationally recognized and widely used ACI Building Code [9].

## 5. Conclusions

The effects of high exposure temperatures on the CT values of two types of carbon steel rebars were investigated. Four different exposure temperatures (20 °C (68 °F), 35 °C (95 °F), 50 °C (122 °F), and 65 °C (149 °C)) at a constant relative humidity of 80% were considered. For each temperature, six different levels of added-chloride ions (0.00%, 0.15%, 0.30%, 0.60%, 0.90%, and 1.20% by weight of cement) were used. Based on the results obtained from this research, the following conclusions can be drawn:

- The corrosion potential and corrosion rate measurements clearly showed a strong coupled effect of the chloride content and temperature on the de-passivation of steel rebars in concrete, especially at high exposure temperatures.
- The CT values determined based on a corrosion rate of 0.1 µA/cm^2^ were consistently within the range of the corresponding CT values obtained from the half-cell potential based on the ASTM C876 criteria.
- The behaviors and trends shown for specimens from Source A and Source B under increasing temperature and chloride levels indicated limited influence of the rebar source on the de-passivation and CT values of carbon steel rebars.
- Temperature had a clear and significant non-linear influence on the CT values. The CT values (water-soluble chlorides by weight of cement) obtained for Source A based on the corrosion rate for the four investigated temperatures were as follows:
  ○ At temperatures of 20 °C (68 °F) and 35 °C (95 °F), the CT values were almost identical at 0.95% and 96%, respectively.
  ○ At 50 °C (122 °F), the CT value decreased to 0.71%.
  ○ At 65 °C (149 °F), the CT value dropped dramatically to 0.26%.

A similar trend and values were obtained for rebars from Source B.

- The effect of increased temperature on the CT value was limited up to a temperature of 35 °C (95 °F). However, a dramatic drop to approximately 1/4 of the CT value at 20 °C (68 °F) was observed at an exposure temperature of 65 °C (149 °F), highlighting the crucial influence of high temperature on the de-passivation of carbon steel rebars in the presence of chlorides.
- The low CT value for carbon steel at an exposure temperature of 65 °C (149 °F) should be a major concern when selecting and specifying durability requirements for RC structures to prevent chloride-induced corrosion under tropical conditions.
- The relevant code provisions for the durability of RC construction in tropical climates need to be assessed considering the exposure class and the effect of high temperature.

## Figures and Tables

**Table 1:** Mixture proportions for materials used in casting concrete specimens.

Materials	Proportions (kg/m^3^)	Proportions (lb/ft^3^)
Cement	350	21.8
Water	175	10.9
Coarse Aggregate	1040	64.9
Crushed Sand	210	13.1
White Sand	490	30.6

**Table 2:** Elemental compositions of steel rebars from two sources (A & B).

Element (%)	C	Mn	Si	Cu	Cr	Ni	Ti	Fe
Source A	0.22	0.59	0.17	-	-	-	-	balance
Source B	0.12	0.85	0.21	0.26	0.07	0.10	0.02	balance

**Table 3:** Water-soluble and acid-soluble Cl ions for different concrete mixes with varying percentages of added-chloride ions.

Added Cl (% wt of Cement)	Water-Soluble Cl (% wt of Cement)	Acid-Soluble Cl (% wt of Cement)	Bound Chloride Percentage
0.00	0.07	0.10	30.0
0.15	0.20	0.25	20.0
0.30	0.33	0.40	17.5
0.60	0.58	0.69	15.9
0.90	0.83	0.97	14.4
1.20	1.12	1.28	12.5

**Table 4:** Assessment of corrosion conditions according to RILEM criteria [37].

Corrosion Condition	I_corr_
Passive condition	<0.1 μA/cm^2^
Low–moderate	0.1 to 0.5 μA/cm^2^
Intermediate–high	0.5 to 1 μA/cm^2^
Very high	>1 μA/cm^2^

**Table 5:** Water-soluble CT values based on measurements of both corrosion potential and corrosion rate.

Exposure Temperature	CT Value (% by Weight of Cement)			
	Half-Cell Potential		Corrosion Rate	
	Source A	Source B	Source A	Source B
20 °C (68 °F)	0.67–1.10	0.90–1.13	0.95	1.12
35 °C (95 °F)	0.75–1.08	0.85–1.15	0.96	1.12
50 °C (122 °F)	0.53–0.88	0.60–0.90	0.71	0.75
65 °C (149 °F)	0.15–0.82	0.15–0.55	0.26	0.25

## Data Availability

Not applicable.
